# Recent decline in prostate cancer incidence in the United States, by age, stage, and Gleason score

**DOI:** 10.1002/cam4.549

**Published:** 2015-12-02

**Authors:** Kimberly A. Herget, Darshan P. Patel, Heidi A. Hanson, Carol Sweeney, William T. Lowrance

**Affiliations:** ^1^ Utah Cancer RegistryUniversity of UtahSalt Lake CityUtah; ^2^ Division of UrologyDepartment of SurgeryUniversity of UtahSalt Lake CityUtah; ^3^ Huntsman Cancer InstituteSalt Lake CityUtah; ^4^ Division of Public HealthDepartment of Family and Preventive MedicineUniversity of UtahSalt Lake CityUtah; ^5^ Division of EpidemiologyDepartment of Internal MedicineUniversity of UtahSalt Lake CityUtah

**Keywords:** Early detection of cancer, incidence, prostate specific antigen, prostatic neoplasms, SEER program, trends

## Abstract

Prostate cancer incidence is sensitive to screening practices, however the impact of recent screening recommendations from the United States Preventative Services Task Force on prostate cancer incidence by age, stage, race, and Gleason score is unknown. This study described the timing and magnitude of changes in prostate cancer incidence trends in the United States by month of diagnosis, and evaluated trends by age, Gleason score, and stage at diagnosis. We analyzed prostate cancer incidence trends using Surveillance, Epidemiology, and End Results (SEER) program data for men diagnosed with invasive prostate cancer from 2007 through 2012. JoinPoint analysis was used to detect changes in the rate of annual percent change (APC) in prostate cancer incidence for all diagnoses and by age, Gleason score, race, and stage. Prostate cancer incidence declined at an estimated −19.6% APC beginning May 2011. This decline was observed in all age groups. Low‐grade tumors (Gleason score ≤6) showed a steeper decline (−29.1% APC) than high‐grade tumors (Gleason score 8–10: −10.8% APC). Only stage I/II and stage III tumors saw declines (−24.2% and −16.7% APC, respectively). A sharp decline in prostate cancer incidence began before release of the United States Preventative Services Task Force October 2011 draft and May 2012 final screening recommendation. The greatest change occurred with incidence of low‐grade tumors, although there is concern that some high‐grade tumors may now go undetected.

## Introduction

Prostate cancer is the most commonly diagnosed malignancy in American men with incidence trends being sensitive to screening 1. Following the introduction of prostate specific antigen (PSA) screening for prostate cancer in the 1980's, the incidence rate increased to a peak of 237 per 100,000 per year among U.S. males in 1992 and has gradually declined since that time.

In 2008, the United States Preventive Services Task Force (USPSTF) issued a Grade D recommendation (recommend against) for PSA screening in men 75 years of age and older 2. In 2009, the Prostate, Lung, Colorectal, and Ovarian (PLCO) Cancer Screening Trial reported no difference in prostate cancer‐specific mortality in screened and unscreened men and the European Randomized Study of Screening for Prostate Cancer (ERSPC) found a small benefit for routine PSA screening 3, 4. Subsequently, the USPSTF issued a grade D recommendation regarding PSA screening in men of all ages, considering the limited benefit of and potential risks associated with prostate biopsy and cancer overtreatment 5. This USPSTF recommendation was presented in a draft document in October 2011 and final recommendation released May 2012. Previous studies have predicted decline of localized prostate cancers 6 and the long‐term risk of an increase of metastatic prostate cancer 7 following the discontinuation of PSA screening.

The U.S. Surveillance, Epidemiology, and End Results (SEER) program, based on data through 2012 and using year as the unit of time for trend analysis, reported trends of prostate cancer incidence declining in the SEER 9 regions by −1.8% per year annual percent change (APC) from 2000 to 2010 and by −12.5% APC from 2010 to 2012 8. In 2007, the prostate cancer rate was 174.9 and it dropped down to 114.1 in 2012 in the SEER 9 regions 8. While there has been a significant decline in prostate cancer incidence, the full impact of the PLCO and ERSPC findings and recent USPSTF recommendations on prostate cancer incidence rates remains uncertain. Recent incidence trends have not been reported according to age at diagnosis, Gleason score, or stage at diagnosis, characteristics that are highly relevant in considering the impact of evolving screening practices. Precise timing of the change in trends may be better understood by evaluating a unit of time smaller than 1 year, as annual rates can mask the within‐year trends 9. We sought to describe the timing and magnitude of changes in prostate cancer incidence trends by month of diagnosis, and to evaluate trends by age, Gleason score, and stage at diagnosis.

## Methods

We identified all malignant prostate cancer cases diagnosed in the SEER 18 registry areas 1 January 2007 through 31 December 2012 using SEER*Stat 10. We excluded all cases with an unknown age at diagnosis or unknown month of diagnosis. We used 2007 as a start point to estimate trends prior to the 2008 recommendation. At the time of this data analysis, 2012 was the most recent year considered complete for cancer surveillance. Because of an interest in the precise timing of any change in incidence trend, we estimated monthly incidence rather than annual incidence rates. Monthly age‐standardized incidence rates were calculated in a manner similar to SEER*Stat method for annual age‐standardized incidence rates. Rather than using the number of cases diagnosed within a year and the annual population counts, we calculated the monthly age‐standardized incidence using the number of new cases diagnosed within a given month as the numerator and the monthly population based on the population year of diagnosis as the denominator.

Trends in prostate cancer incidence over time were analyzed using JoinPoint software 11. JoinPoint finds between one and four straight line segments that best fit the shape of the data. Using a Monte Carlo Permutation method 12, the program tests which number of line segments is the closest to the time pattern of the data. The JoinPoint model also estimates the percent change per unit time in each segment. All APC values were tested against the hypothesis that the slope was equal to zero, using a two sided test based on a t‐distribution and an alpha of *P* < 0.05.

A JoinPoint model was fit for overall prostate cancer incidence, and then separate models were fit for subgroups by age at diagnosis: (45–54, 55–64, 65–74, and 75 and older). We estimated incidence of prostate cancer by grade as defined by Gleason score: low grade (Gleason score ≤6), intermediate grade (Gleason score = 7), and high grade (Gleason score = 8, 9, or 10). For stage at diagnosis, we used AJCC TNM Classification and Stage Groupings, 6th edition 13, and classified tumors into groups: I/II, III, IV, and unknown. Stage I and II were combined due to the small number of prostate cancer cases diagnosed at stage I. For race and ethnicity, we used the following groups: non‐Hispanic Whites, Hispanic (any race), Black (non‐Hispanic), American Indian or Alaskan Natives (non‐Hispanic), and Asian or Pacific Islander (non‐Hispanic). The number of JoinPoint segments for these sub‐analyses were restricted to not exceed the number detected in the overall analysis.

## Results

**Figure 1:**
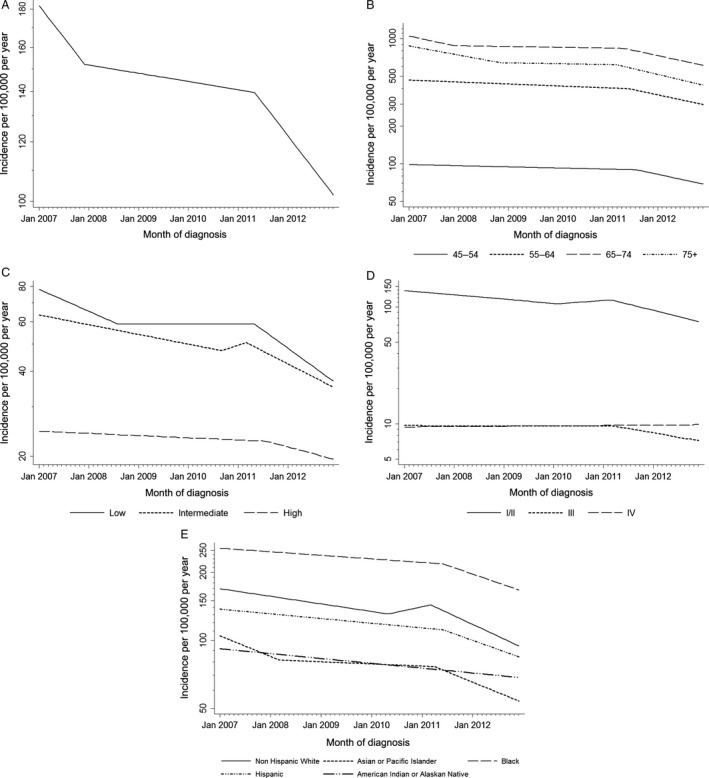
Recent trends in prostate cancer incidence in Surveillance, Epidemiology, and End Results 18, JoinPoint regression by month of diagnosis. (A) Prostate cancer incidence, age‐adjusted. (B) Incidence by age at diagnosis. (C) Incidence of low‐ (Gleason score ≤6), intermediate‐ (Gleason score 7) and high‐grade (Gleason score 8–10) prostate tumors, age‐adjusted. (D) Incidence by stage at diagnosis age‐adjusted. (E) Incidence by race and ethnicity, age‐adjusted. Inflection points represent time points of significant change in trend.

There were 349,517 prostate cancer cases diagnosed in the 18 SEER registries from 2007 to 2012 (Table 1). Gleason score was unknown for 6.5% of cases and stage was unknown for 7.7%. For the trend analysis, we excluded 71 cases with unknown age and 3300 cases with an unknown month of diagnosis (<1% of the total), resulting in 346,217 cases for analysis (Table 1). The JoinPoint analysis detected two significant changes in prostate cancer incidence trends during this period (Fig. 1A). The first was in December 2007, from −19.4% APC to −2.5% APC and the second in May 2011, when the incidence rate began another steep decline, −19.6% APC (Table 2).

**Table 1:** Prostate cancer cases reported by 18 Surveillance, Epidemiology, and End Results registries, 2007–2012

	*N*	%
Total cases^a^	346,217	
Year of diagnosis		
2007	61,935	17.9
2008	58,621	16.9
2009	59,163	17.1
2010	58,017	16.8
2011	58,647	16.9
2012	49,834	14.4
Age at diagnosis		
Under 45	2085	0.6
45–54 years	33,575	9.7
55–64 years	113,412	32.8
65–74 years	126,878	36.6
75 or older	70,267	20.3
Gleason score		
Low (≤6)	146,524	42.3
Intermediate (7)	124,564	36.0
High (8–10)	52,503	15.2
Unknown	22,626	6.5
Stage at diagnosis^b^		
I/II	272,934	78.8
III	24,435	7.1
IV	22,226	6.4
Unknown	26,622	7.7
Race and Ethnicity		
White^c^	238,148	71.1
Hispanic (any race)	30,315	9.1
Black^c^	49,673	14.8
American Indian or Alaska Native^c^	1156	0.3
Asian or Pacific Islander^c^	15,578	4.7

a Excluding cases with unknown month of diagnosis.

b Based on American Joint Commission on Cancer's Classification and Stage groupings, 6th edition.

c Excluding Hispanics.

**Table 2:** Recent trends in prostate cancer incidence in 18 Surveillance, Epidemiology, and End Results Registries 2007–2012, JoinPoint regression by month of diagnosis

	Trend 1		JoinPoint 1	Trend 2		JoinPoint 2	Trend 3	
	Annual percent change	95% confidence interval	Month of change	Annual percent change	95% confidence interval	Month of change	Annual percent change	95% confidence interval
Overall	−19.4^c^	−33.1, −5.5	December 2007	−2.5^c^	−4.6, −0.4	May 2011	−19.6^c^	−26.3, −12.9
Age at diagnosis								
45–54	−2.1^c^	−3.5, −0.7	August 2011	−19.4	−29.1, −9.7			
55–64	−3.6^c^	−4.9, −2.2	June 2011	−19.2	−26.4, −12.0			
65–74	−19.3^c^	−34.1, −4.4	December 2007	−1.5	−3.7, 0.8	May 2011	−19.5^c^	−26.4, −12.4
75+	−16.8^c^	−22.9, −10.6	November 2008	−1.5	−6.4, 3.4	March 2011	−21.3^c^	−29.1, −13.6
Gleason score								
Low	−17.8^c^	−24.1, −11.3	August 2008	0.0	−3.1, 3.1	May 2011	−29.1^c^	−36.2, −21.9
Intermediate	−7.9^c^	−9.9, −5.9	September 2010	13.1	−42.2, 70.9	March 2011	−20.5^c^	−27.0, −14.0
High	−1.8^c^	−3.3, −0.3	August 2011	−10.8^c^	−20.7, −0.8			
Unknown	1.2	−0.9, 3.3	None					
Stage at diagnosis^a^								
I/II	−8.4^c^	−10.8, −6.0	February 2010	7.0	−6.1, 20.2	March 2011	−24.2^c^	−30.1, −18.2
III	−0.1	−2.0, 1.8	March 2011	−16.7^c^	−23.8, −9.4			
IV	0.7	−0.3, 1.7	None					
Unknown	−5.3^c^	−7.0, −3.7	None					
Race and Ethnicity								
White^b^	−7.7^c^	−9.9, −5.6	May 2010	11.3	−9.8, 32.8	March 2011	−23.8^c^	−29.9, −17.6
Hispanic (any race)	−4.8^c^	−6.7, −2.9	June 2011	−18.2^c^	−28.2, −8.1			
Black^b^	−3.6^c^	−5.2, −2.1	June 2011	−17.6^c^	−25.8, −9.3			
American Indian or Alaska Native^b^	−4.9^c^	−8.4, −1.5	None					
Asian or Pacific Islander^b^	−20.9^c^	−34.0, −7.7	March 2008	−2.2	−5.5, 1.1	April 2011	−20.9^c^	−28.9, −12.8

a Based on American Joint Commission on Cancer's Classification and Stage groupings, 6th edition.

b Excluding Hispanics.

c Annual percent change significantly differs from zero, *P* < 0.05.

Prostate cancer incidence for men aged 75 and older decreased steeply (−16.8% APC) from January 2007 to November 2008 before stabilizing for several years. The rate started dropping again in March 2011 (−21.3% APC). Similarly for men aged 65–74, the incidence rate dropped steeply from January 2007 to December 2007 (−19.3% APC) and then stabilized before dropping again after May 2011 (−19.5% APC) (Fig. 1B). For the younger two age groups, there was a small decline from 2007 to 2011, followed by a steep decline starting in June 2011 for those aged 55–64 (−19.2% APC) and August 2011 for those aged 45–54 (−19.4% APC).

The incidence of low Gleason score cancers declined, −17.8% APC, from January 2007 through August 2008 (Fig. 1C), stabilized, and then dropped (−29.1% APC, beginning in May 2011). Incidence of intermediate‐grade prostate cancer decreased at −7.9% per year from January 2007 to September 2010, then increased non‐significantly, followed by a sharp decline, (−20.5% APC), beginning in March 2011. Incidence of high‐grade prostate cancer declined slowly, −1.8% APC, from January 2007 until August 2011, and was followed by a steep decline of −10.8% APC. Considering the most recent trend beginning in 2011, the APCs for low‐grade and high‐grade prostate were significantly different from each other.

For stage at diagnosis, the stage I and II cancers declined moderately (−8.4% APC) from January 2007 to February 2010 (Fig. 1D). Beginning in March 2011, the rate declined at a much faster rate (−24.2% APC). There was little change in the incidence of stage III prostate cancer until March 2011, when it started to decline (−16.7% APC) at a slower rate than the stage I/II cancers. The incidence of late‐stage disease did not change during the observed period (0.7% APC). The incidence of unknown cancers declined at a constant rate over the observed period (−5.3% APC).

When trends were examined by race and ethnicity, we found that prostate cancer incidence as decreased for all groups over time (Fig. 1E). The steepest decline in incidence was after 2011. Non‐Hispanic whites had the earliest (March 2011) and steepest (−23.8% APC) change in rates. They were followed by Asian and Pacific Islanders (April 2011, −20.9% APC). Incidence rates for Hispanics and Blacks both declined starting in June 2011, but the decline was not as steep as non‐Hispanic whites (−18.2% APC and −17.6% APC respectively). These differences in declines were not statistically significant. American Indian and Alaskan Natives had a small, steady decline (−4.9% APC) over the whole study period.

## Discussion

Our analysis of prostate cancer incidence trends by month of diagnosis, age, grade and stage found the change in screening recommendations was followed by a steep decline in prostate cancer incidence, as predicted by previous studies 6, 7. The best estimate of the timing of the start of the recent steep decline in prostate cancer incidence was in May 2011, before release of the draft recommendation by the USPSTF but after publication of the PLCO and ERSPC results.

The goal of prostate cancer screening is to detect high‐risk tumors while they are treatable and potentially curable. Unfortunately, prostate cancer screening strategies with PSA and digital rectal exam are not specific for high‐risk disease and often lead to diagnosis and overtreatment of lower risk tumors that may not impact survival. In our analyses, the steepest reduction in the 2011–2012 time period was for low‐grade tumors and for cancers in stage I or II. Because men diagnosed with low‐grade tumors and early‐stage cancers have a low prostate cancer‐specific mortality, the decline in their incidence reflects reduced detection of tumors with little benefit from diagnosis and treatment relative to risks ratio 14. However, some of cancers with higher prostate cancer‐specific mortality, high‐grade prostate cancer and stage III cancers, also declined in 2011–2012.

The slopes for the decline beginning in 2011 were similar for all age groups. When the entire study period (2007–2012) is considered, the greatest decline in prostate cancer incidence was seen in men 75 and older, although the differences between the age groups were not statistically significant. This is consistent with the earlier release of the 2008 USPSTF recommendation discouraging screening for this age group. However, this decline is not as drastic until after March 2011, which further expands the trends that have been seen in SEER data through 2009 by age group that suggested an effect of the 2008 USPSTF recommendations 15. It also supports that previous studies have shown varying levels of impact of USPSTF recommendations on prostate cancer screening recommendations given by primary care providers to their patients 15, 16, 17, 18, 19.

Racial and ethnic differences in the decline of prostate cancer incidence may be due to differences in PSA screening or mortality. Non‐Hispanic white men and black men have historically had higher rates of PSA screening compared to Hispanics or other races 20, and therefore it is not surprising that non‐Hispanic white men had the largest rate of decline. Additionally, prostate cancer incidence and mortality is significantly higher in black men 8 and black men are diagnosed at a younger age and have traditionally had higher PSA levels at diagnosis 21. Following the 2012 guideline change, non‐Hispanic white males were the only racial group to significantly decrease their rates of PSA testing 22. The drop in PSA screening among white men combined with the higher risk profile for black men may be leading to the small racial differences observed here.

Trend analyses are sensitive to the selection of time intervals, and trends may be attenuated if there are not enough data points. The SEER program‐reported trend of −12.5% for 2010–2012 was detected by JoinPoint based on three annual incidence estimates, the minimum number of data points required to describe a trend 2. Our analysis by month estimated the trend to be steeper, −19.6% APC, and to start later, in May 2011. The two analyses also differ in the numbers of registries included and ranges of years examined. Our estimates of monthly prostate cancer incidence may be imprecise due to reliance on population denominators that were only available annually. However, the impact should be minimal as population growth in the SEER 18 catchment areas was <1% per year over the time period examined. This analysis is based on descriptive epidemiology data representing 28% of the U.S. population. Changes in cancer incidence trends in general are affected not only by screening patterns but also by changes in cancer risk factors and population demographics. However, we believe that changes in risk factors or population demographics are less likely explanations for the observed short‐term steep decline in prostate cancer incidence than changes in screening.

One report has documented a decline in newly diagnosed low‐risk and high‐risk prostate cancer cases after the recommendation change 23, however, this analysis was limited to cancer counts rather than incidence rates, and was not population‐based. Using population‐based cancer registry data, the present study documents the changes in prostate cancer incidence trends coinciding with the release of the PLCO and ERSPC studies and recent USPSTF PSA screening recommendations by month of diagnosis. Decreasing detection of low‐grade prostate cancer in elderly men will likely have a positive public health impact, but there is concern that some high‐grade tumors will go undetected with evolving PSA screening practices. Since prostate cancer incidence data are available only through 2012, additional surveillance data are needed to determine the long‐term impact of declining PSA screening on prostate cancer diagnosis and mortality, particularly with regard to the incidence rate of stage IV cancers.

## Conflict of Interest

None declared.

## References

[1] Siegel, R. L. , K. D. Miller , and A. Jemal . 2015 Cancer statistics, 2015. CA Cancer J. Clin. 65:5–29. 25559415 10.3322/caac.21254

[2] 2008 Screening for prostate cancer: U.S. Preventive Services Task Force recommendation statement. Ann. Intern. Med. 149:185–191. 18678845 10.7326/0003-4819-149-3-200808050-00008

[3] Schroder, F. H. , J. Hugosson , M. J. Roobol , T. L. J. Tammela , S. Ciatto , V. Nelen , et al. 2009 Screening and prostate‐cancer mortality in a randomized European study. N. Engl. J. Med. 360:1320–1328. 19297566 10.1056/NEJMoa0810084

[4] Andriole, G. L. , E. D. Crawford , R. L. Grubb III , S.S. Buys , D. Chia , T. R. Church , et al. 2009 Mortality results from a randomized prostate‐cancer screening trial. N. Engl. J. Med. 360:1310–1319. 19297565 10.1056/NEJMoa0810696 PMC2944770

[5] Moyer, V. A. ; Force USPST . 2012 Screening for prostate cancer: U.S. Preventive Services Task Force recommendation statement. Ann. Intern. Med. 157:120–134. 22801674 10.7326/0003-4819-157-2-201207170-00459

[6] Gulati, R. , A. Tsodikov , R. Etzioni , R. A. Hunter‐Merrill , J. L. Gore , A. B. Mariotto , et al. 2014 Expected population impacts of discontinued prostate‐specific antigen screening. Cancer 120:3519–3526. 25065910 10.1002/cncr.28932 PMC4221407

[7] Scosyrev, E. , G. Wu , S. Mohile , and E. M. Messing . 2012 Prostate‐specific antigen screening for prostate cancer and the risk of overt metastatic disease at presentation: analysis of trends over time. Cancer 118:5768–5776. 22847578 10.1002/cncr.27503

[8] Howlader N. N. A., Krapcho M., Garshell J., Miller D., Altekruse S. F., Kosary C. L., et al., eds. 2015 SEER cancer statistics review, 1975–2012. National Cancer Institute, Bethesda, MD.

[9] Ravdin, P. M. , K. A. Cronin , N. Howlader , C. D. Berg , R. T. Chlebowski , E. J. Feuer , et al. 2007 The decrease in breast‐cancer incidence in 2003 in the United States. N. Engl. J. Med. 356:1670–1674. 17442911 10.1056/NEJMsr070105

[10] Surveillance, Epidemiology, and End Results (SEER) Program (www.seer.cancer.gov) SEER*Stat Database. Incidence—SEER 18 Regs Research Data + Hurricane Katrina Impacted Louisiana Cases, Nov 2014 Sub (2000‐2012) <Katrina/Rita Population Adjustment>—Linked To County Attributes—Total U.S., 1969‐2013 Counties. In: National Cancer Institute DCCPS, Surveillance Research Program, Surveillance Systems Branch, released April 2015, based on the November 2014 submission.

[11] 2011 Joinpoint Regression Program, Version 3.5.2. Statistical Research and Applications Branch, National Cancer Institute, Bethesda, MD.

[12] Kim, H. J. , M. P. Fay , E. J. Feuer , and D. N. Midthune . 2000 Permutation tests for joinpoint regression with applications to cancer rates. Stat. Med. 19:335–351. 10649300 10.1002/(sici)1097-0258(20000215)19:3<335::aid-sim336>3.0.co;2-z

[13] Green F. L., Page D. L., Fleming I. D., Fritz A. G., Balch C. M., Haller D. G., et al., eds. 2002 American Joint Committee on cancer staging manual, 6th ed Springer, New York.

[14] Klotz, L. , D. Vesprini , P. Sethukavalan , V. Jethava , L. Zhang , S. Jain , et al. 2015 Long‐term follow‐up of a large active surveillance cohort of patients with prostate cancer. J. Clin. Oncol. 33:272–277. 25512465 10.1200/JCO.2014.55.1192

[15] Howard, D. H. 2012 Declines in prostate cancer incidence after changes in screening recommendations. Arch. Intern. Med. 172:1267–1268. 22826149 10.1001/archinternmed.2012.2768

[16] Prasad, S. M. , M. W. Drazer , D. Huo , J. C. Hu , and S. E. Eggener . 2012 2008 US Preventive Services Task Force recommendations and prostate cancer screening rates. JAMA 307:1692–1694. 22535850 10.1001/jama.2012.534

[17] Howard, D. H. , F. K. Tangka , G. P. Guy , D. U. Ekwueme , and J. Lipscomb . 2013 Prostate cancer screening in men ages 75 and older fell by 8 percentage points after Task Force recommendation. Health Aff. (Millwood) 32:596–602. 23459740 10.1377/hlthaff.2012.0555 PMC4818375

[18] Goodwin, J. S. , A. Tan , E. Jaramillo , and Y. F. Kuo . 2013 Prostate‐specific antigen testing in men aged 40‐64 years: impact of publication of clinical trials. J. Natl. Cancer Inst. 105:743–745. 23459245 10.1093/jnci/djt039 PMC3716227

[19] Ross, J. S. , R. Wang , J. B. Long , C. P. Gross , and X. Ma . 2012 Impact of the 2008 US Preventive Services Task Force recommendation to discontinue prostate cancer screening among male Medicare beneficiaries. Arch. Intern. Med. 172:1601–1603. 22987029 10.1001/archinternmed.2012.3726 PMC3597450

[20] Ross, L. E. , Y. J. Taylor , L. C. Richardson , and D. L. Howard . 2009 Patterns in prostate‐specific antigen test use and digital rectal examinations in the Behavioral Risk Factor Surveillance System, 2002–2006. J. Natl. Med. Assoc. 101:316–324. 19397221 10.1016/s0027-9684(15)30878-6

[21] Shao, Y. H. , K. Demissie , W. Shih , A. R. Mehta , M. N. Stein , C. B. Roberts , et al. 2009 Contemporary risk profile of prostate cancer in the United States. J. Natl. Cancer Inst. 101:1280–1283. 19713548 10.1093/jnci/djp262 PMC2744729

[22] Li, J. , Z. Berkowitz , and I. J. Hall . 2015 Decrease in Prostate Cancer Testing Following the US Preventive Services Task Force (USPSTF) Recommendations. J. Am. Board Fam. Med. 28:491–493. 26152440 10.3122/jabfm.2015.04.150062 PMC6077843

[23] Barocas, D. A. , K. Mallin , A. J. Graves , D. F. Penson , B. Palis , D. P. Winchester , et al. 2015 The effect of the United States Preventive Services Task Force grade D recommendation against screening for prostate cancer on incident prostate cancer diagnoses in the US. J. Urol. [Epub ahead of print]. 10.1016/j.juro.2015.06.075 26087383

